# Correction to: LINC01296/miR-26a/GALNT3 axis contributes to colorectal cancer progression by regulating *O*-glycosylated MUC1 via PI3K/AKT pathway

**DOI:** 10.1186/s13046-019-1140-0

**Published:** 2019-04-01

**Authors:** Bing Liu, Shimeng Pan, Yang Xiao, Qianqian Liu, Jingchao Xu, Li Jia

**Affiliations:** 1 College of Laboratory Medicine, Dalian Medical University, 9 Lushunnan Road Xiduan, Dalian, 116044 Liaoning Province China; 2 Department of General Surgery, the Second Affiliated Hospital of Dalian Medical University, Dalian, 116027 Liaoning Province China

**Correction to: J Exp Clin Cancer Res (2018) 37:316**

**https://doi.org/10.1186/s13046-018-0994-x**

In the publication of this article [1], there is an error in Fig. 5G (panel 2, group of InmiR-26a + siSCR in HCT-8/5-FU, treated with 284.3 μM). The revised Fig. 5 which includes 5G has now been included in this correction.

**Fig. 5:**
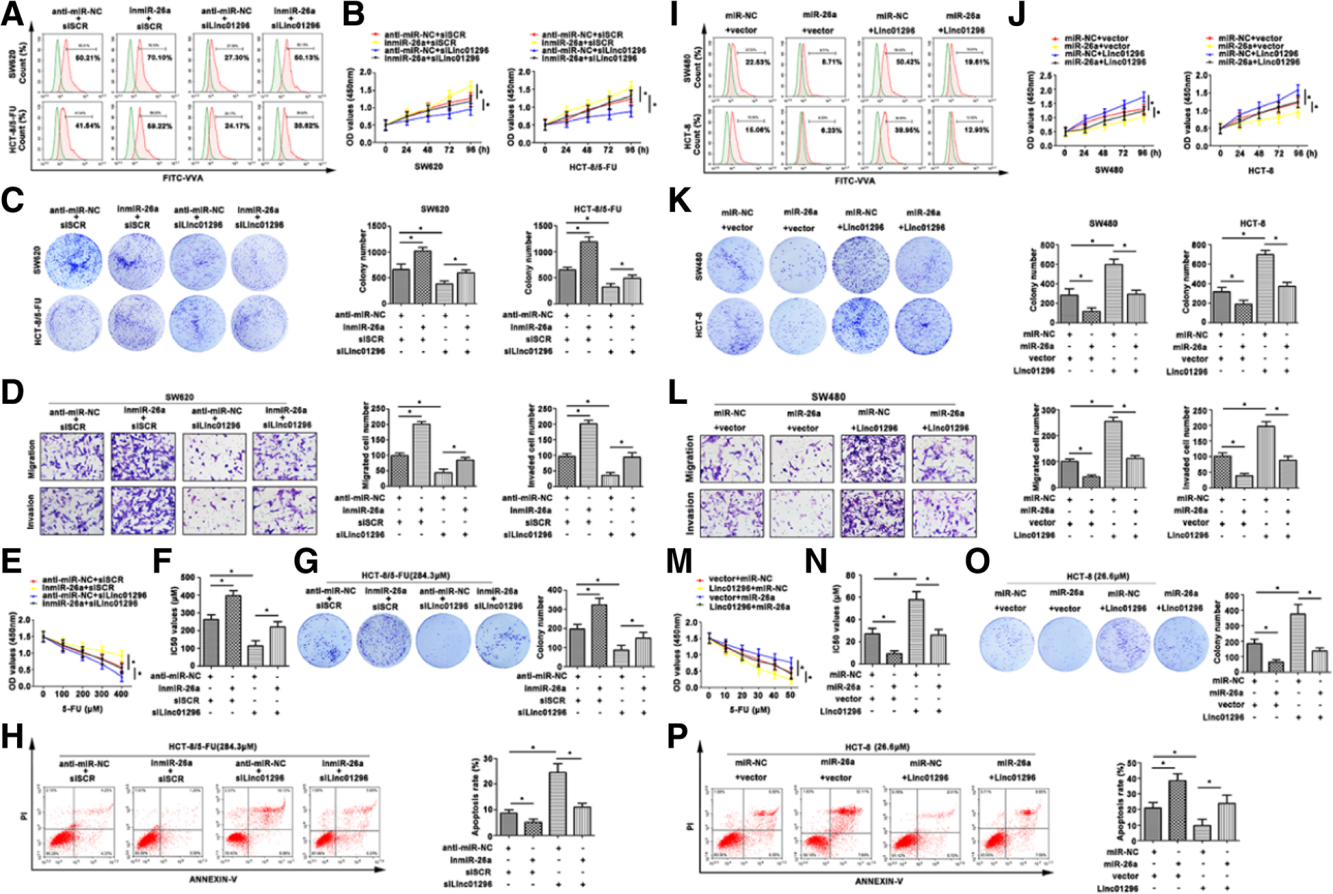
The effect of co-expression linc01296 and miR-26a on CRC progression. **a** Flow cytometry showed O-linked glycosylation level detected by fluorescein isothiocyanate (FITC)-conjugated VVA on the cell surface of transfected CRC cells. **b** CCK8 assay showed variant growth rate of transfected CRC cells. **c** Colony formation assay detected proliferative formation with different treated CRC cells. **d** Aggressiveness changed was determined by transwell assay, the migratory and invasive cells were counted. **e** CRC cells were treated with 5-FU, and the cell viability was detected by CCK8 assay. **f** IC50 values of treated CRC cells were calculated. **g** The effect of 5-FU on CRC cell proliferation was determined by colony formation assay. **h** Flow cytometry showed the apoptosis rate of transfected CRC cells in response to 5-FU. **i** FITC-VVA on transfected CRC cells surface was detected by flow cytometry. **j** The growth curves of transfected CRC cells were pictured after conducting CCK8 assay. **k** Proliferation of treated CRC cells was examined by colony formation assay. **l** Migration and invasion were observed in transfected SW620 cells. **m** With 5-FU treatment, the cell viability was determined by CCK8 assay. **n** The IC50 values were subsequently calculated. **o** 5-FU resistant CRC cells were transfected with linc01296 or miR-26a mimic, and the colony formation was counted in response to 5-FU. **p** The apoptosis rate of transfected CRC cells was detected after 5-FU treatment by flow cytometry. The data were means ± SD of three independent assays (*P < 0.05)

The correct Fig. 5 is given hereafter:

This error does not affect discussions and conclusions drawn in the article.
